# Retraction: Histone Deacetylase Inhibitors Sensitize Lung Cancer Cells to Hyperthermia: Involvement of Ku70/SirT-1 in Thermo-Protection

**DOI:** 10.1371/journal.pone.0237589

**Published:** 2020-08-06

**Authors:** The PLOS ONE Editors

After this article [1] was published, concerns were raised about several of the reported results.

Specifically:

- Similarities were noted between the following panels of Fig 1A and S1 Fig. These two figures report results of experiments using different cell lines.
  ○ Fig 1A No treatment, S1 Fig No treatment, and S1 Fig Hyperthermia only
  ○ Fig 1A NM only and S1 Fig NAM only
  ○ Fig 1A TSA only and S1 Fig TsA only
  ○ Fig 1A TSA+NAM only and S1 Fig NAM+TsA only
  ○ Fig 1A TSA+NAM+hyperthermia and S1 Fig NAM+hyperthermia
  ○ S1 Fig TsA+hyperthermia and S1 Fig NAM+TsA+hyperthermia
    The authors provided some image data to support these experiments and commented that the PC-10 results had been shown in S1 Fig in error. For several panels, there were discrepancies in data labels provided in the published figures versus in the image data file provided post-publication, and as such, questions about the reported results remain unresolved for both the PC-10 and H1299 experiments. Furthermore, based on the data received, it is unclear whether the results reported for H1299 cells were supported by the data.
- In Fig 1, no loading control data are reported for the Bax western blot experiment in panel C, and panel D (described in the legend) is missing from the figure. An author commented that some proteins commonly used as loading controls (e.g. actin and tubulin) may be affected by hyperthermia and so they routinely use CBB or Ponceau S staining to visualize protein levels. Loading control data for the Fig 1C experiment are not available. Data were not provided to support the claims made in the article based on the missing Fig 1D panel.
- Similarities were noted between the following western blot panels:
  ○ Fig 2A Bax lanes 1–4, which reports a western blot using whole cell extracts as input, and Fig 3A Bax, which reports an immunoprecipitation-western blot experiment.
  ○ Fig 2A Bclx_L_ (lanes 2, 3) and S4 Fig Bclx_L_.
  ○ Fig 2A Bcl2, lanes 4 and 5 appear similar and there appears to be a vertical discontinuity near the left side of lane 5.
  ○ Fig 2A Ku70 (lanes 2–5) and Fig 4A AntiKu70 Ab (W.B.). In response to this, the authors noted that the same data applied in the two experiments. However, different timepoints are indicated for these data in the two figures.
  ○ Fig 2A Ku70 (lanes 4–5) and S4 Fig Ku70; these data represent results for different cell lines and different time points.
  ○ Fig 2B Bcl2/40 μg panel and S4 Fig Bcl2.
  ○ Fig 2B Bclx_L_ panels for 20 and 40 μg.
  ○ In the Fig 4C Ku70 panel, lanes 2 and 3 appear similar, when aspect ratio is adjusted.
  ○ In the Fig 6A Actin panel, lanes 1–3 appear similar to lanes 4–6 when rotated 180 degrees. The author commented that this may have been due to an error.
- In Fig 2A, lanes 2 and 3 of the Ponceau S panel appear similar. A corresponding author commented that these similarities are due to similarities in loading. However, per our editorial assessment, the image data in the indicated lanes are more similar than would be expected for different experimental results. In addition, lanes 1, 2, 4, and 5 of the Fig 2A Ponceau S panel appear similar to lanes 1–4, respectively, of the Fig 7B Control siRNA Ponceau S panel. These represent different experiments.
- In Fig 3B, there appears to be a vertical discontinuity suggestive of image splicing after lane 4 of the Ku70 blot, and there is no visible image data in lane 5 of this blot. The author confirmed that there is no image data in lane 5 of the blots in this figure (except for Bax) but did not clarify whether the figure includes negative data for 6-h (IgG) samples, or whether 6-h (IgG) data were not included in the Ku70 and Ku80 panels.
- In S6 Fig, there appear to be several vertical and/or horizontal discontinuities suggestive of image splicing, and in the IP:Bax Ab, Bax panel there appears to be a box overlay in lane 5 that is discontinuous with the rest of the panel image. The authors commented that images were spliced between lanes 4 and 5 in preparing the figures; they did not comment on the other visible discontinuities.
- The backgrounds of several western blot panels reported in the article appear uniform and/or adjusted such that one cannot distinguish features in the background image. A corresponding author commented that the quality of the reported images may have been affected by the imaging technique: according to the author, results were photographed using polaroid cameras and then scanned to obtain digital images.

A corresponding author commented on the concerns and in some cases disagreed with the issues noted. Supporting data from related or replicate experiments were provided in response to some of the concerns, but the author noted that the original data underlying results reported in [1] are not available, except for the microscopy images provided in support of Fig 1A and S1 Fig. Without the original data, the image concerns cannot be clarified.

In light of the above concerns which call into question the reliability of the reported results, the *PLOS ONE* Editors retract this article.

HW, YF, YO and NS agreed with retraction. A-eS-e, ASS, and ZM either could not be reached or did not respond directly. MKH disagreed with the retraction and noted that he stands by the findings reported in the article.
